# Is there association between chronic kidney disease and dental caries? A case-controlled study

**DOI:** 10.4317/medoral.22737

**Published:** 2019-03

**Authors:** Cláudia RSD Menezes, Antônio LA Pereira, Cecília CC Ribeiro, Cláudia O Chaves, Rosane NM Guerra, Érika BAF Thomaz, Valério Monteiro-Neto, Cláudia MC Alves

**Affiliations:** 1 PhD in Collective Health - Federal University of Maranhão, São Luís – MA, Brazil; 2 PhD, Postgraduate Program of Dentistry, Federal University of Maranhão, São Luís - MA, Brazil; 3 PhD, Pathology Department – Federal University of Maranhão, São Luís - MA, Brazil; 4 PhD, Postgraduate Program of Public Health, Federal University of Maranhão, São Luís - MA, Brazil; 5 PhD, Laboratory of Microbiology, CEUMA University; São Luís - MA, Brazil

## Abstract

**Background:**

The purpose of this study was to assess the association between chronic kidney diseases (CKD) and dental caries.

**Material and Methods:**

107 patients with CKD and 107 with no systemic alteration were randomly included. DMFT (decayed, missing, and filled teeth), plaque index, colony-forming units (CFU) of Streptococcus mutans and salivary composition (IgA total, IgA anti- Streptococcus mutans, calcium and urea) were evaluated. McNemar and Wilcoxon tests were used to compare test and control groups. Spearman test was used to correlate time of hemodialysis and variables studied. Associations between variables were evaluated by logistic regression analysis.

**Results:**

The number of filled teeth, the amount of IgA anti-Streptococcus mutans, salivary urea, education level, monthly income and the amount of CFU of Streptococcus mutans were statistically different between groups. There was a positive correlation between the duration of hemodialysis (Hd) and the amount of IgA anti-Streptococcus mutans, urea in saliva, and the number of CFU of Streptococcus mutans. In the adjusted model, a higher incidence of CFU mutans streptococci, elevated salivary urea, smaller number of filled teeth, lower DMFT, and less calcium salivary were associated with CKD.

**Conclusions:**

Programs to prevent and treat oral problems and regular follow-up at the beginning of dialysis are necessary to increase patients’ awareness of their condition.

** Key words:**Renal disease, chronic, dental caries, renal dialysis.

## Introduction

Chronic Kidney Disease (CKD) is characterized by irregularities in the structure and renal function for 3 months or more. It is classified in 5 stages based on the decrease of the glomerular filtration rate and the increase in the rate of albuminuria that can lead to end-stage of renal disease (ESRD) (1). Dialysis and renal transplantation are treatments indicated for patients in ESRD (2).

Oral complications have been observed in CKD patients, such as changes in salivary composition (3) including elevated levels of urea (4), potassium and phosphate and reduced levels of calcium (5,6), reduced salivary flow (7,8), salivary pH, which tends to be more alkaline (4,9), increased salivary buffering capacity (9-11), and increased dental calculus formation (4,5).

However, no consensus exists regarding the presence of dental caries. Some investigators did not observe difference in the number of decayed, missing or filled teeth (DMFT) when comparing adult CKD patients and healthy subjects (12,13). Others have found a higher DMFT index in case groups (14-16). Low dental caries indices (5,11,16,17) and higher DMTF levels in CKD patients on dialysis have been observed though (8). Also, a high plaque index (PI) has been demonstrated in patients with CKD (11-13,16).

 High concentration of urea in saliva may induce in CKD patients the formation of calculus and uremic breath (8), but also contribute to the remineralization of dental enamel, leading to a lower occurrence of caries (5). In addition, elevated salivary urea may alter the oral environment, reducing *Streptococcus mutans* and lactobacilli in children (11,15).

Thus, considering the lack of consensus regarding the predisposition of patients with CKD to the development of dental caries, the objective of the present study was to assess the association between ESRD and dental caries.

## Material and Methods

-Selection of patients 

This is a case-controlled study nested to a cross-sectional study. The study was approved by the Ethics Committee of Hospital Universitário – Unidade Presidente Dutra (HUPD) (process 33104-037/205), São Luis, MA, Brazil. All patients received detailed information about the research and signed a free informed consent to participate in the study.

The case group (n = 107) was randomly selected among 453 patients of both genders, ranging in age from 20 to 87 years old with an average of 44.64 years old in the end-stage renal disease who were undergoing Hd at three referral centers, 115 at Pro-Renal, 262 at Nephrology and Arterial Hypertension Center (CENEFRON) and 76 at HUPD.

The exclusion criteria were: edentulous patients; patients on Hd for less than 3 months, having other concomitant systemic diseases, such as diabetes, lupus erythematosus and amyloidosis, and who had received antibiotic therapy during the 3 months prior to saliva collection. Before examination, patients answered a questionnaire, including complete name, address, gender, age, educational level and income, systemic condition, oral hygiene habits, dental visit frequency and duration of the hemodialysis.

Control group consisted of 107 subjects with no systemic alteration matched for gender and age range to the case group. The average age of the group was 43.97 years old. They were recruited among individuals seen at the Dental Health Service of Hospital Aderson de Sousa Lopes (HASL), according to the order of admission to the hospital.

-Clinical, microbiological and salivary evaluation

The presence of dental caries was evaluated using the DMFT index (18). Clinical exam was conducted using a periodontal probe PCPUNC 156 (Hu-Friedy, Chicago, USA) and an oral mirror under artificial light with the patient sitting in the Hd chair. Plaque index (PI) or presence of biofilm was evaluated according to the presence of visible biofilm in the mesial, buccal and lingual surfaces of all teeth (19).

Non-stimulated saliva samples were collected from 100 patients. The 50 patients of the study group were selected randomly, and 50 participants from the control group were frequency matched for gender and age range to the study group. Saliva was collected over a period of 5 to 10 min and stored in individual, sterile, glass containers. After collection, the material was immediately submitted to microbiological analysis and frozen for IgA titration and evaluation of salivary composition. The total number of colony-forming units (CFU/mL saliva) of *Streptococcus mutans* was determined using the Dentalcut II kit (Laborclin, Pinhais, Brazil) after incubation at 35º C for 24-48 hrs. The number of CFU/mL was estimated according to manufacturer instructions.

Salivary composition was evaluated by the determination of total IgA, anti-*Streptococcus mutans* IgA, calcium and urea. The total IgA and anti-*Streptococcus mutans* IgA were assayed by ELISA® (Enzyme-Linked Immunosorbent Assay) as previously described (20). Calcium was determined by spectrophotometry at 570 nm using the Calcium Liquiform kit (Labtest, Lagoa Santa, Brazil). Salivary urea was quantified at 340 nm using the Urea UV Liquiform kit (Labtest, Lagoa Santa, Brazil).

-Statistical analysis

Statistical analysis was performed with the STATA® program (StataCorp, College Station, TX, USA). Descriptive statistics were made through absolute frequencies, averages and standard deviations. The Shapiro-Wilk test was used to assess the distribution of the data. As the data showed a non-normal distribution, non-parametric tests were used to assess whether there were statistically significant differences between groups.

The McNemar and Wilcoxon tests were used for comparison of variables between the case and control groups. The Spearman correlation test was used to evaluate correlations between the duration of Hd and oral health-related variables. Associations between the variables were evaluated by logistic regression analysis. The crude, unadjusted, univariate and adjusted, multivariate odds ratios (OR) were estimated. Variables with *P* values less than 20% in the crude analysis were retained in the multivariable model to adjust. The significance level was set at 5%.

## Results

The distribution of variables for both studied groups is shown in  Table 1. Statistical hypothesis testing demonstrated that case presented lower educational level (*p* = 0.04), lower monthly income (*p* = 0.01), greater presence of CFU of Streptococcus mutans in saliva (*p* = 0.02), fewer teeth filled (*p*<0.001) and higher amounts of anti-mutans IgA (*p*=0.04) and urea in the saliva (*p*<0.001) compared to controls.

**Table 1:**
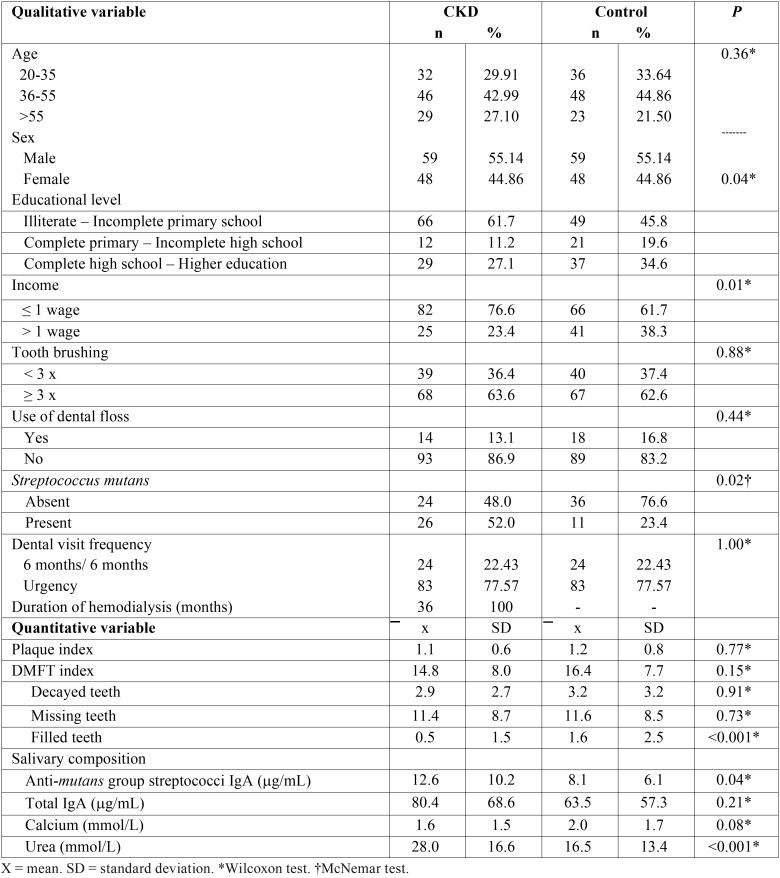
Descriptive data obtained for patients with CKD and controls.

The univariate regression analysis (crude model) revealed a possible association (*p* ≤ 0.2) between ESRD and some variables that were maintained in the multivariate model (adjusted model). In the final model, only the presence of *Streptococcus mutans* (OR = 3.55; IC = 1.05-11.9; *p* = 0.04), lower DMFT (OR = 0.92; IC = 0.85-0.98; *p* = 0.02), smaller number of filled teeth (OR = 0.70; IC = 0.51-0.97; *p* = 0.03), lower concentration of salivary calcium (OR = 0.58; IC = 0.37-0.90; p = 0.01) and higher concentration of salivary urea (OR =1.07; IC = 1.02-1.13; *p* <0.05) remained associated with ESRD ( Table 2).

**Table 2:**
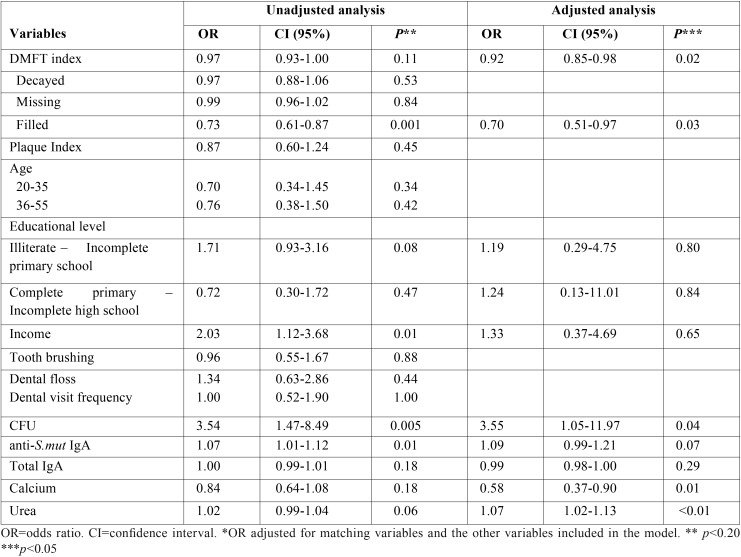
Association between socioeconomic and oral health variables and CKD.

Additionally, there was a positive correlation between the duration of hemodialysis and the quantity of IgA anti-*Streptococcus mutans* (r = 0.25; *p* = 0.01), urea in saliva (r = 0.42; *p* < 0.01), the number of CFU of *Streptococcus mutans* (r = 0.22; *p* < 0.05). The number of filled teeth had a negative correlation with duration of hemodialysis (r = -0.30; *p* < 0.001) (data not shown).

## Discussion

In the present investigation, an association was observed between ESRD on hemodialysis and a greater isolation of CFU in *Streptococcus mutans*, elevated concentration of salivary urea, reduced concentration of salivary calcium, reduced DMFT index and smaller amount of filled teeth ( Table 2).

Data on oral health parameters of CKD patients have been controversial. Some studies reported no difference in PI between patients with ESRD and controls (12-15), whereas others showed higher average plaque index in ESRD patients on dialysis (13,21,22). Several factors may have contributed to these differences, including inadequate adjusting for some confounding variables.

In this research, PI was similar in case and control groups. Considering that oral hygiene habits and the frequency of visits to the dentist were similar in the two groups, this result is plausible. Although there are differences between the groups studied in terms of education and monthly income, these two factors do not appear to have affected oral hygiene habits and attendance to the dentist, as ESRD was not related to those variables.

Differences in the mean DMFT index were not observed between groups ( Table 1). Similar (12,13), low (5,10,17) and high (14,21) DMFT indices between case and control groups have been reported in literature. Such divergence can also be influenced by multiple factors as discussed above, including time of renal disease. There was no correlation between the DMFT and the duration of dialysis in this study.

Another important factor to be considered is the age of the subjects in previous studies compared to our research, as lowest average DMFT index were observed in children and adolescents (10,17). In the present study, although statistical tests did not show significant differences when comparing average DMFT indices, regression analysis showed a slight association of ESRD with a smaller DMFT. None of the evaluated studies conducted association analysis on their data using multiple regressions.

Patients with ESRD presented a significantly smaller number of filled teeth in this paper ( Table 1), according to data of previous research with similar sample (23). A possible explanation is that most (77.6%) of these patients only seek dental care in case of pain or for tooth extraction. In addition, many professionals avoid attending patients with systemic diseases because of apprehension, lack of preparation or lack of infrastructure (23).

The level of salivary calcium was similar in both the case and control groups, contrasting with former data (5). However, an association between ESRD and lower levels of salivary calcium was observed, which could be explained by vitamin D deficiency, which is common in patients with renal deficiency and compromises calcium homeostasis (24). Moreover, it is known that this ion is important to the remineralizing capacity of saliva (25).

Also, a significantly high concentration of salivary urea was observed in patients with ESRD, in agreement with previous studies (4,5). Elevated salivary urea levels have been suggested as a mechanism that protects the tooth against demineralization (5,6), which render the salivary pH alkaline that is kept even after dialysis (5).

A significantly high frequency of isolation of *Strep. mutans* in patients with ESRD was observed in this study. These findings contrast with previous studies (11,15). The level of salivary urea might not have been high enough to suppress bacterial growth as previously suggested (15). Furthermore, in those previous studies Streptococcus mutans counts were determined in children (11,15) and no study in adult patients was found in the literature.

Although higher *Streptococcus mutans* counts were observed in patients with ESRD the level of colonization was not indicative of a high caries risk. One possible explanation for this finding is the relatively small size of the sample, which increases variance and reduces the precision of the estimates.

No studies evaluating IgA anti-mutans in patients with CKD were found in the literature. Analysis of the IgA anti-mutans titers suggests that higher quantities of this specific antibody could also serve as a protective factor against dental caries in this study. Also, salivary IgA anti-mutans and urea levels were found to be significantly high in patients receiving Hd therapy for a long period of time.

There was a negative correlation between the number of filled teeth and the duration of hemodialysis. Some studies in the literature have shown a positive correlation between the duration of Hd, DMFT index and its components (26) and PI (16,26).

Possible explanations for these divergences can be pointed out, as the age range and hemodialysis time of the populations. In one investigation, the sample consisted of children, adolescents and young adults. The mean age was therefore lower than that of the present series (mean = 44.3 years, SD = 14.9), a factor increasing the differences in caries experience of the study samples and impairing eventual comparisons (16).

The present study showed that patients with ESRD on Hd are comparable to systemically healthy subjects in terms of the PI and prevalence of dental caries. However, differences in the number of filled teeth, colonization with *Streptococcus mutans* and salivary IgA anti-mutans and urea levels were observed between patients with ESRD and controls. Those associations reinforce the importance of studies, as the amount of salivary urea represented a protective factor for tooth decay and calcium level and the isolation frequency of *Streptococcus mutans* represented a risk factor for tooth decay in patients with CKD.

Moreover, in the present study, control subjects were recruited among patients seen at a dental clinic, a fact that might have biased the associations in this study in the direction of the null hypothesis. Longitudinal, multicenter studies could better assess the effect of CKD on the occurrence of tooth decay.

In view of the debilitated systemic condition of ESRD patients on Hd, the implementation of programs for the prevention and treatment of oral problems as well as regular follow-up at the beginning of dialysis are necessary to increase the patient’s awareness regarding his condition, since an acceptable oral health status is required for the execution and maintenance of a possible kidney transplant.
